# Tuning Atomically Dispersed Fe Sites in Metal–Organic Frameworks Boosts Peroxidase-Like Activity for Sensitive Biosensing

**DOI:** 10.1007/s40820-020-00520-3

**Published:** 2020-09-23

**Authors:** Weiqing Xu, Yikun Kang, Lei Jiao, Yu Wu, Hongye Yan, Jinli Li, Wenling Gu, Weiyu Song, Chengzhou Zhu

**Affiliations:** 1 Key Laboratory of Pesticide and Chemical Biology of Ministry of Education, International Joint Research Center for Intelligent Biosensing Technology and Health, College of Chemistry, Central China Normal University, Wuhan, 430079 People’s Republic of China; 2 State Key Laboratory of Heavy Oil Processing, China University of Petroleum, Beijing, 102249 People’s Republic of China

**Keywords:** Nanozymes, Metal–organic frameworks, Atomically dispersed sites, Peroxidase-like activity, Biosensors

## Abstract

Although nanozymes have been widely developed, accurate design of highly active sites at the atomic level to mimic the electronic and geometrical structure of enzymes and the exploration of underlying mechanisms still face significant challenges. Herein, two functional groups with opposite electron modulation abilities (nitro and amino) were introduced into the metal–organic frameworks (MIL-101(Fe)) to tune the atomically dispersed metal sites and thus regulate the enzyme-like activity. Notably, the functionalization of nitro can enhance the peroxidase (POD)-like activity of MIL-101(Fe), while the amino is poles apart. Theoretical calculations demonstrate that the introduction of nitro can not only regulate the geometry of adsorbed intermediates but also improve the electronic structure of metal active sites. Benefiting from both geometric and electronic effects, the nitro-functionalized MIL-101(Fe) with a low reaction energy barrier for the HO* formation exhibits a superior POD-like activity. As a concept of the application, a nitro-functionalized MIL-101(Fe)-based biosensor was elaborately applied for the sensitive detection of acetylcholinesterase activity in the range of 0.2–50 mU mL^−1^ with a limit of detection of 0.14 mU mL^−1^. Moreover, the detection of organophosphorus pesticides was also achieved. This work not only opens up new prospects for the rational design of highly active nanozymes at the atomic scale but also enhances the performance of nanozyme-based biosensors.

**Electronic supplementary material:**

The online version of this article (10.1007/s40820-020-00520-3) contains supplementary material, which is available to authorized users.

## Highlights

- The two functional groups (nitro and amino) were introduced into MIL-101(Fe) for tuning the atomically dispersed metal active sites.
- Benefiting from both geometric and electronic effects, the nitro-functionalized MIL-101(Fe) shows a superior electronic structure of active sites and low reaction energy barrier for the HO* formation.
- Nitro-functionalized MIL-101(Fe)-based biosensor was successfully employed to detect acetylcholinesterase activity and organophosphorus pesticide.

**Electronic supplementary material:**

The online version of this article (10.1007/s40820-020-00520-3) contains supplementary material, which is available to authorized users.

## Graphical abstract

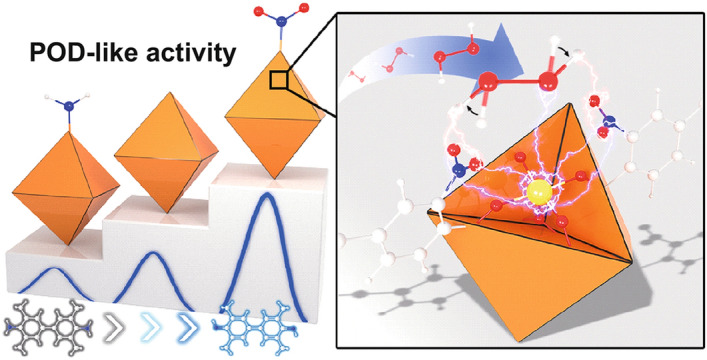

## Introduction

Peroxidase (POD) with superior catalytic activity and selectivity has been widely applied in the fields of analytical sensing [1, 2]. The active site of POD contains a penta-coordinate heme iron, where adjacent atoms can directly affect the electronic structure of the central Fe site and contribute to the activity and selectivity [3, 4]. Nevertheless, the main challenge in natural enzymes is poor stability, which limits the activity and lifetime of enzymes in complex environments [5, 6]. Recently, nanozymes, a series of nanomaterials with enzyme-mimicking characteristics, have been selected as the substitutes for natural enzymes and applied in various fields because of their high stability, low-cost and mass production [7–11]. However, the catalytic activities of nanozymes are still much lower than those of natural enzymes [12–14]. Given the electronic and geometrical structures of enzymes are two key factors toward catalytic activities, accurately designing and tuning the electronic and geometrical structure of active sites at the atomic scale are important for the development of advanced nanozymes [15–18]. Specifically, the well-defined structures and coordination features of atomically dispersed active sites are of great significance for understanding the activity–structure relationships in nanozyme systems and achieving vivid mimicking of natural enzymes [19–22].

Metal–organic frameworks (MOFs) have received widespread applications in various fields because of their attractive features including large surface area, high porosity, structural diversity and functional tunability [23–26]. Significantly by virtue of the versatile organic linkers and atomically dispersed metal structural building units, the microstructure and the electronic structure of MOFs could be well regulated via various strategies [27–31]. In particular, the introduction of functional groups or heteroatoms in the MOFs can not only affect the electron density around the atomically dispersed metal centers but also change the nucleophilicity, redox potential and stability of the catalysts, making them own great potential in electrocatalysis, photocatalysis and biocatalysis [32–36]. Recently, the enzyme-like property of MOFs has attracted the extensive interest of the research community. However, their catalytic activities are still less than satisfactory compared with other types of nanozymes [37–40]. Therefore, the regulation of active sites based on geometric and electronic effects has great potential for the enhancement of catalytic activities of nanozymes.

Herein, MIL-101(Fe) (abbreviated as MIL-101 here) with the favorable POD-like activity was chosen as the model, where the Fe sites coordinate with five neighbor atoms as the active sites of natural POD. Benefiting from predictable and designable functionality, two functional groups, nitro and amino (–NO_2_ and –NH_2_) with opposite electron modulation abilities, are controllably introduced into MIL-101 (denoted as NO_2_-MIL-101 and NH_2_-MIL-101, respectively) to tune the structure of active sites. As expected, the functional groups can efficiently regulate POD-like activity. The NO_2_-MIL-101 exhibited superior POD-like performance, followed by MIL-101 and finally NH_2_-MIL-101. The experiment results demonstrate that the introduction of –NO_2_ efficiently enhances the affinity of MIL-101 toward the substrate, which dramatically promotes its catalytic activity. Furthermore, theoretical studies indicate that the strong electron-withdrawing –NO_2_ can not only regulate the geometry of the adsorbed intermediates but also efficiently optimize the electronic structure of atomically dispersed Fe, leading to a significant reduction of energy barrier for the HO* formation and the enhancement of POD-like activity. Therefore, tuning atomically dispersed Fe sites in MOFs with synergistic geometric and electronic effects provides great opportunities to design advanced nanozymes and improve the catalytic activity at the atomic scale. As a concept application, the NO_2_-MIL-101-based biosensor was established for sensitive colorimetric determination of acetylcholinesterase (AChE) activity and organophosphorus pesticides (OP), which holds great promise in biosensing applications.

## Experimental

### Materials

Acetylcholinesterase (AChE), acetylthiocholine chloride (ATCh), 5,5-dimethyl-1-pyrroline 1-oxide (DMPO), invertase (INV) and bovine serum albumin (BSA) were purchased from Sigma-Aldrich. 3,3′,5,5′-tetramethylbenzidine (TMB) was obtained from Shanghai Dibai Biotechnology Co., Ltd. Dihydroethidium (DHE), glucose oxidase (GOx), alkaline phosphatase (ALP), laccase (LAC), horseradish peroxidase (HRP), 2-nitroterephthalic acid, terephthalic acid, and 2-aminoterephthalic acid were from Shanghai Aladdin Bio-Chem Technology Co., Ltd. Paraoxon-ethyl and other pesticides were from Dr. Ehrenstorfer GmbH (Augsburg, Germany). All the chemical reagents obtained were of analytical reagent grade.

### Evaluation of the POD-Like Activity

The nanozymes (1 mg mL^−1^, 10 µL) were introduced into the HAc-NaAc buffer (0.1 M, pH 3.0, 150 µL) containing H_2_O_2_ (100 mM, 100 µL) and TMB (1 mM, 50 µL). Then, the absorbance values of the reaction solution were obtained by a multimode reader after 5 min.

### Kinetics Assay

The kinetics assay of the nanozymes evaluated the catalytic ability of nanozymes in different concentrations of TMB and H_2_O_2_. In brief, the nanozymes (1 mg mL^−1^, 10 µL) were added into the HAc-NaAc buffer (pH 3.0, 150 µL) containing H_2_O_2_ (0.5 M, 100 µL) and different concentrations of TMB (50 µL) to obtain the kinetic data toward TMB. Similarly, the nanozymes (1 mg mL^−1^, 10 µL) were added into the HAc-NaAc buffer (pH 3.0, 150 µL) containing TMB (10 mM, 50 µL) and different concentrations of H_2_O_2_ (100 µL) to obtain the kinetic data toward H_2_O_2_. Then, a typical Michaelis–Menten equation (*V* = *V*_max_[*S*]/(*K*_*m*_ + [*S*])) was used to evaluate the kinetics of nanozymes. The *V* is the velocity, *V*_max_ is the maximal reaction velocity, [*S*] is the substrate concentration, and *K*_*m*_ is the Michaelis–Menten constant.

### Computation Details

Density functional theory (DFT) calculations were performed by Vienna Ab initio Simulation Package (VASP) with the generalized gradient approximation (GGA) parameterized by Perdew, Burke and Ernzerhof (PBE) for the exchange correlation functional [41–43]. We used a local structural unit of MIL-101 with three equivalent Fe atoms to simulate the unmodified MIL-101. We replaced the hydrogen with six amino groups and six nitro groups at the same site to simulate the NH_2_-MIL-101 and NO_2_-MIL-101, respectively. We used a 30 × 30 × 30 Å^3^ cubic lattice to prevent spurious interactions due to periodic boundary conditions. An energy cutoff of 300 eV is used for all calculations, and the *k*-point meshes of 1 × 1 × 1 were applied for Brillouin zone integration. The atomic positions were relaxed until the force on each atom was less than 0.05 eV Å^−1^, and the convergence tolerance of the energy was set to be 10^−4^ eV.

### Colorimetric Detection of AChE

ATCh (10 mM, 30 µL, pH 7.4) and different concentrations of AChE (50 µL, pH 7.4) were incubated for 30 min 37 °C. Then, the NO_2_-MIL-101 (1 mg mL^−1^, 20 µL), HAc-NaAc buffer (0.1 M, pH 3.0, 1 mL) and H_2_O_2_ (100 mM, 200 µL) were introduced into this reaction solution. After incubation for another 10 min, TMB (1 mM, 200 µL) was introduced into this system. The absorbance of this mixture (named *A*) was recorded after 5 min. A control group without the addition of ATCh was also carried out to obtain *A*_0_. A linear relationship between the (*A*_0_ − *A*)/*A*_0_ × 100 and activity of AChE was obtained for the evaluation of AChE activity.

## Results and Discussion

### Synthesis and Characterization of MOFs

**Fig. 1:**
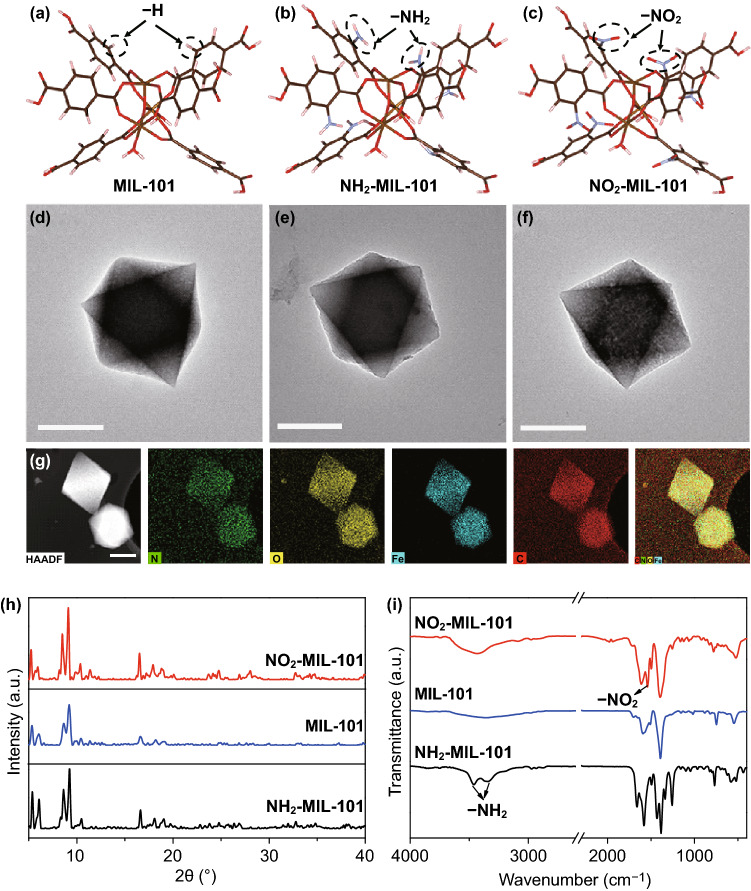
Structures of **a** MIL-101, **b** NH_2_-MIL-101, and **c** NO_2_-MIL-101 (Fe: yellow, C: brown, O: red, H: pink, N: purple). TEM images of **d** MIL-101, **e** NH_2_-MIL-101, and **f** NO_2_-MIL-101. **g** HAADF-STEM image and STEM-EDS mappings of NO_2_-MIL-101, scale bar: 500 nm. **h** XRD patterns and **i** FT-IR spectra of MIL-101, NH_2_-MIL-101 and NO_2_-MIL-101. (Color figure online)

The MIL-101 with POD-like characteristics, excellent stability and functional tunability was synthesized by using Fe^3+^ as a coordination center and terephthalic acid as organic ligands [44], which is selected as the model to design the modified nanozymes. To regulate the electronic or microstructure of MOFs, the strong electron-withdrawing –NO_2_ and electron-donating –NH_2_ groups are introduced to synthesize functionalized MIL-101, respectively. The detailed synthetic procedures are presented in Supporting Information. It is established that the nodes of MIL-101 are constituted by three octahedra sharing a *µ*_3_-O corner and one Fe at the center [45]. As shown in Fig. 1a–c, the theoretical models of the local structural unit of the different functionalized MIL-101 are the same, except that the –NH_2_ or –NO_2_ replaces the –H of terephthalic acid. First, the morphologies of the as-prepared materials were characterized by transmission electron microscopy (TEM). As can be seen in Fig. 1d–f, the MIL-101, NO_2_-MIL-101 and NH_2_-MIL-101 show uniform octahedron morphologies with a diameter of about 600–800 nm. High-angle annular dark-field scanning TEM (HADDF-STEM) image and its corresponding energy-dispersive spectroscopy (STEM-EDS) mappings demonstrate the uniform dispersion of C, N, O and Fe in NO_2_-MIL-101 (Fig. 1g). Moreover, the powder X-ray diffraction (XRD) patterns suggest that the MIL-101, NH_2_-MIL-101 and NO_2_-MIL-101 share a similar topology (Fig. 1h). Also, compared with the Fourier transform infrared spectroscopy (FT-IR) spectra of MIL-101, the presence of characteristic peaks of –NH_2_ and –NO_2_ in NH_2_-MIL-101 and NO_2_-MIL-101, respectively, verify that the –NH_2_ and –NO_2_ have been successfully introduced into the MIL-101 (Fig. 1i). Moreover, X-ray photoelectron spectroscopy (XPS) was performed to further verify the elemental compositions of the resultant MOFs. The peaks of C 1s, O 1s and Fe 2p were observed in the full-range XPS spectra (Fig. S1a). The significant peak of N 1s in the XPS spectra manifests the existence of –NH_2_ (399.67 eV) and –NO_2_ (405.52 eV), which were well consistent with the observation from FT-IR (Fig. S1b) [46, 47].

### POD-Like Activity of MOFs

**Fig. 2:**
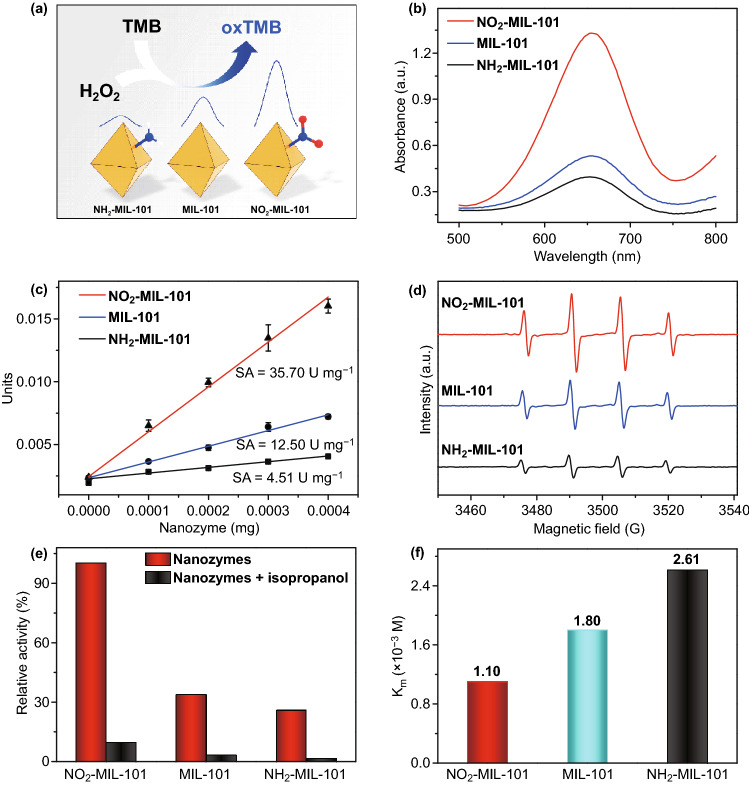
**a** Schematic diagram of the MOF-based nanozymes to mimic POD. **b** Absorption spectra of TMB catalyzed by the different nanozymes in the system containing H_2_O_2_ and HAc-NaAc (pH = 3.0). **c** Specific activities of nanozymes. **d** EPR spectra of different nanozymes in the system containing H_2_O_2_, 5,5-dimethyl-1-pyrroline 1-oxide (DMPO) and HAc-NaAc (pH = 3.0). **e** Relative activity of nanozymes in the presence and absence of isopropanol. **f** K_m_ of nanozymes for H_2_O_2_

The introduction of the functional groups in MIL-101 was expected to rationally tune the POD-like activity. Based on the same content of metal active sites (Fe) (Table S1), a typical chromogenic reaction was conducted, in which the colorless 3,3′,5,5′-tetramethylbenzidine (TMB) was oxidized to the blue oxidation state of TMB (oxTMB) in the presence of H_2_O_2_ and nanozymes (Fig. 2a). It is found that the introduction of electron-withdrawing –NO_2_ enhances the POD-like activity of MIL-101, while the introduction of electron-donating –NH_2_ weakens the POD-like activity of MIL-101 (Fig. 2b). The specific activity (SA) was used to quantitatively evaluate the catalytic activity of these nanozymes under the optimal condition (pH = 3.0, Fig. S2) [48]. Figure 2c shows that the SA of the NO_2_-MIL-101 and MIL-101 is 7.91- and 2.77-fold higher than that of NH_2_-MIL-101, respectively. Then, the electron paramagnetic resonance (EPR) spectroscopy verified the existence of hydroxyl radicals (·OH) (Fig. 2d), and the intensities of the signal peaks reflecting the relative content of ·OH are in good consistency with their POD-like activities [49]. The same conclusions were reached by using the degradation rates of methylene blue to indirectly monitoring the concentration of ·OH (Fig. S3) [50]. Moreover, the catalytic activities of these nanozymes were dramatically decreased upon the addition of isopropanol, which is a scavenger agent for ·OH [51], indicating that the ·OH was the main active intermediate (Fig. 2e). Then, the steady-state kinetics of these nanozymes was investigated to probe the enhanced POD-like activity of NO_2_-MIL-101 (Fig. S4-S6). The Michaelis–Menten constant (*K*_*m*_) value of NO_2_-MIL-101 for H_2_O_2_ is lower than that of the other nanozymes, indicating the NO_2_-MIL-101 has a higher affinity toward H_2_O_2_ than that of the other nanozymes (Fig. 2f). Furthermore, the *V*_max_ values of NO_2_-MIL-101 for both H_2_O_2_ and TMB are the highest among these nanozymes (Table S2). Based on these results, the introduction of –NO_2_ is noteworthy to improve the POD-like activity of MIL-101, while the introduction of –NH_2_ exhibits the opposite effect. Notably, the excellent thermostability and reproducibility of the three MOFs were further verified, holding great promise for practical applications (Fig. S7).

### Mechanisms for POD-Like Activity of MOFs

**Fig. 3:**
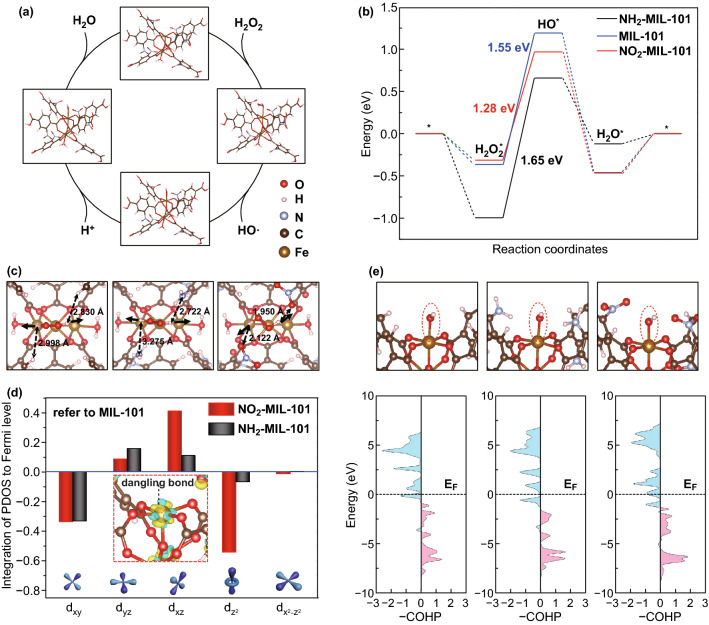
**a** Schematic diagram of the elementary step of reaction in an acidic environment on NO_2_-MIL-101. **b** Energy change diagram of reaction on MIL-101, NH_2_-MIL-101 and NO_2_-MIL-101, respectively. **c** Geometry structure of H_2_O_2_* adsorbed on MIL-101, NH_2_-MIL-101 and NO_2_-MIL-101 (from left to right). The solid arrows represent the orientation of the O–H bond, and the dotted line represents the distance from H on the adsorbed H_2_O_2_ to the ligand. **d** Integration of the PDOS to Fermi level of each split Fe 3d orbit on MIL-101, NH_2_-MIL-101 and NO_2_-MIL-101, where the unmodified MIL-101 is taken as a reference. Inset: the local view near the Fe active site, yellow (blue) isosurfaces denote an increase (decrease) of 0.01 e/Å-3 for electronic density. **e** Schematic diagram of HO*-Fe bond and the corresponding pCOHP (from left to right: MIL-101, NH_2_-MIL-101 and NO_2_-MIL-101, respectively)

To reveal the origin of the outstanding catalytic activity of NO_2_-MIL-101, DFT calculations were used to illustrate the geometric and electronic effects of –NO_2_. The models were established based on the local structural unit of MIL-101 with three Fe atoms, where the upper exposed Fe was employed as the reaction site (*). The reaction mechanism under acidic conditions was followed for calculations, in which the first adsorbed H_2_O_2_* is cleaved into an ·OH and an adsorbed hydroxyl group (HO*) and then a protonated hydrogen approach OH* to form the H_2_O* and subsequently desorb (Fig. 3a and Eqs. S1-S4). As we can see in Fig. 3b, the adsorption of H_2_O_2_* is thermodynamically favorable, as well as the formation and desorption of H_2_O*. Notably, the splitting of H_2_O_2_* (H_2_O_2_* → HO* + ·OH) acted as the rate-determining steps (RDS) on the three investigated models, where the energy change of RDS is NO_2_-MIL-101 (1.28 eV) < MIL-101 (1.55 eV) < NH_2_-MIL-101 (1.65 eV), which is consistent well with the experimental results of catalytic activity test. Obviously, the adsorbed H_2_O_2_* and HO* intermediates play important roles in the reaction. To explore the reason for the superior catalytic activity of NO_2_-MIL-101, we first focus on the geometric effect of –NO_2_ on the adsorbed H_2_O_2_*. Interestingly, on NO_2_-MIL-101, the structure of the adsorbed H_2_O_2_* is different, where the orientation of the O–H bond of H_2_O_2_* biased to the oxygen on the –NO_2_ (Fig. 3c). In contrast, no significant shift was observed on MIL-101 and NH_2_-MIL-101. The horizontal adsorption structure of H_2_O_2_* on NO_2_-MIL-101 caused by orientation shift of the O–H bond shortens the distance between H and –NO_2_ (H···O–N–O) to 1.950 Å and 2.122 Å, respectively, which is ascribed to the strong electron absorption effect of –NO_2_. Notably, this reasonable change in the absorbed H_2_O_2_* structure on NO_2_-MIL-101 is conducive to the breaking of the O–O bond and consequently reducing the energy change of the HO* formation.

To further uncover the effect of the –NO_2_ from the perspective of electronic structure, charge density difference on NO_2_-MIL-101 was calculated for the –NO_2_. Figure S8 depicts the change of electron density when the NO_2_ ligands exist. Obviously, apart from the fact that electrons are localized around the ligands to form the N–C bonds, the electron density at the Fe active site also changes significantly, that is, the electron decreases along the octahedral direction of the Fe–O bond (including the above dangling bond), while increases in the non-bond direction. We know that the $d_{{z^{2} }}$ and $d_{{x^{2} - z^{2} }}$ among the split d orbits are generally attributed to the anti-bond along the octahedral direction (Fe–O in this work) in the octahedral field (usually donated as, eg*), while $d_{xy}$, $d_{xz}$ and $d_{yz}$ are attributed to the non-bond orbit (i.e., the *t*_2g_). To clarify the electron change of atomically disperse Fe, we calculated the project electronic density of states (PDOS) of the splitting 3d orbit ($d_{xy}$, $d_{xz}$, $d_{yz}$, $d_{{z^{2} }}$ and $d_{{x^{2} - z^{2} }}$) of the Fe atoms on each investigated model. And the energy of PDOS was integrated into the Fermi level for each splitting 3*d* orbit to compare the electronic change quantitatively (Table S3). As shown in Fig. 3d, for NO_2_-MIL-101, the integration of PDOS in ${\text{d}}_{\text{xz}}$ direction increased and that in the ${\text{d}}_{{{\text{z}}^{ 2} }}$ direction decreased significantly, while no such a dramatic change occurred in NH_2_-MIL-101. Consequently, we believe that the enhanced activity of NO_2_-MIL-101 may stem from the decreased electrons on the dangling bond (the $d_{{z^{2} }}$ direction) at the Fe active site. For an in-depth understanding of the role of changed 3d orbital split during the catalytic reaction, we calculated the projected Crystal Orbital Hamilton Population (pCOHP) to reveal the interaction between the adsorbed HO* and Fe atom at the active site (Fig. 3e) [52]. Moreover, we computed the integrated COHP (ICOHP) for the HO*–Fe bond on each model to explore the bond strength quantitatively through calculating the energy of pCOHP integral up to the Fermi level (Table S4). As usual, the positive (red area) represents the bond contributions, and the negative (blue area) represents the anti-bond contributions in the pCOHP diagram. The anti-bond orbital filling of the HO*–Fe bond (the blue area below Fermi level) in NO_2_-MIL-101 is less than that in MIL-101 and NH_2_-MIL-101, indicating the electrons in the anti-bond orbit are reduced. Moreover, the ICOHP of HO*-Fe bond on NO_2_-MIL-101 (− 4.80) is more negative than that on MIL-101 (− 4.11) and NH_2_-MIL-101 (− 4.13), meaning that the reduced anti-bonding electrons increase the bond strength of HO*–Fe on NO_2_-MIL-101 compared to the other two nanozymes, which facilitates the cleavage of H_2_O_2_* and consequently reduces the energy change of RDS.

### Construction of Biosensors

**Fig. 4:**
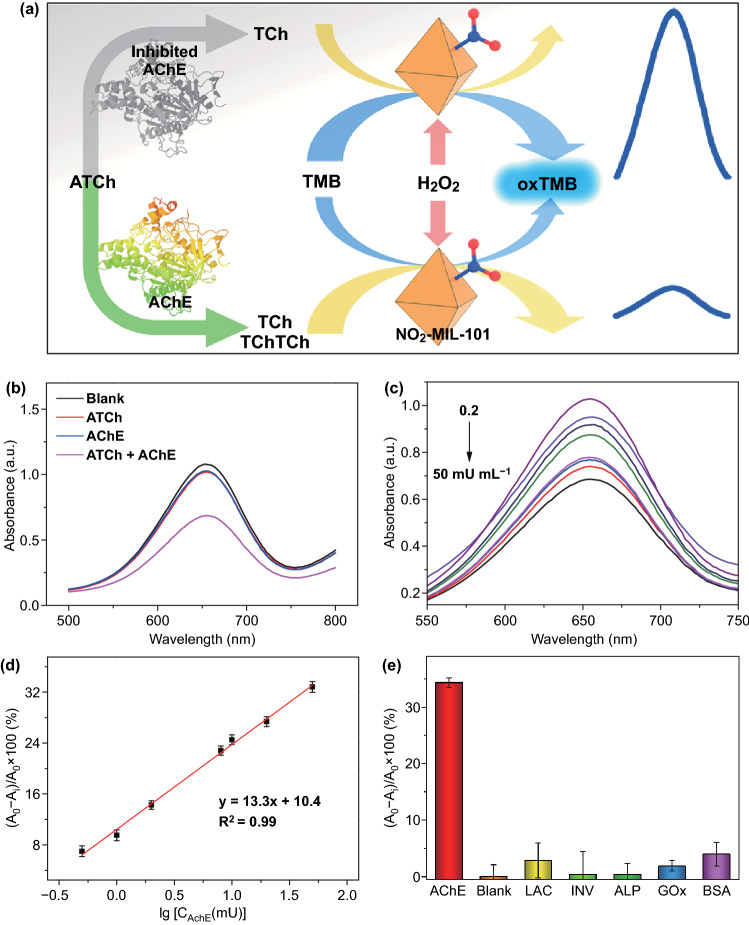
**a** Schematic illustration of detecting AChE activity using a NO_2_-MIL-101-based biosensor. **b** Absorption spectra of ATCh, AChE and ATCh + AChE in the NO_2_-MIL-101-based biosensor. **c** Absorption spectra of NO_2_-MIL-101-based biosensor in the presence of different AChE concentrations. **d** A linear relationship between the AChE activity and the variation of absorbance at 652 nm. **e** Selectivity test of NO_2_-MIL-101-based biosensor in the presence of interfering proteins

AChE, as one kind of important enzymes in biological nerve conduction activity, is wildly used as biomarkers in clinical diagnosis and biosensing areas [53, 54]. Hence, the construction of a simple and highly sensitive biosensor for the detection of the AChE activity is extremely urgent. AChE can specifically hydrolyze acetylthiocholine (ATCh) into thiocholine (TCh), a mercapto molecule, which can inhibit the POD-like performance of nanozymes [55]. Based on this phenomenon, a real-time colorimetric NO_2_-MIL-101-based biosensor was applied to determine AChE activity (Fig. 4a). As shown in Fig. 4b, the extent of oxTMB decreased significantly in the presence of ATCh and AChE. To improve the performance of biosensor, the incubation time, the concentration of nanozymes and the temperature were optimized (Figs. S9 and S10). Under the optimized conditions, with the increasing amounts of AChE, the concentration of oxTMB weakens gradually (Fig. 4c). Accordingly, there is a good linear relationship between the activity of AChE and absorbance values in the range of 0.2–50 mU mL^−1^ with a limit of detection (LOD) of 0.14 mU mL^−1^ (Fig. 4d). Notably, compared with other biosensors reported previously, the resultant biosensor possesses higher sensitivity for the assay of AChE activity (Table S7). Furthermore, various proteins, including horseradish peroxidase (HRP), laccase (LAC), glucose oxidase (GOx), invertase (INV) and bovine serum albumin (BSA), were selected as interferents to estimate the specificity of this biosensor. Although the reductive mercapto functional groups in BSA and LAC can influence the performance of biosensor, resulting in slightly higher response values (Fig. 4e), the inhibitions of these proteins were negligible compared with that of AChE, indicating the satisfactory selectivity of the proposed biosensor. Besides, both the result of parallel experiments at the same time and the good response of this biosensor after stored several days indicate that the developed biosensor exhibits good reproducibility and stability (Figs. S11a and S12a). Furthermore, the AChE-spiked serum samples were used to investigate the feasibility of the developed biosensor in human serum samples. The corresponding recoveries ranged from 96.0 to 102.1%, demonstrating that the proposed biosensors have good feasibility (Table S5). These results indicate that the developed biosensor exhibits great promises in practical application.

**Fig. 5:**
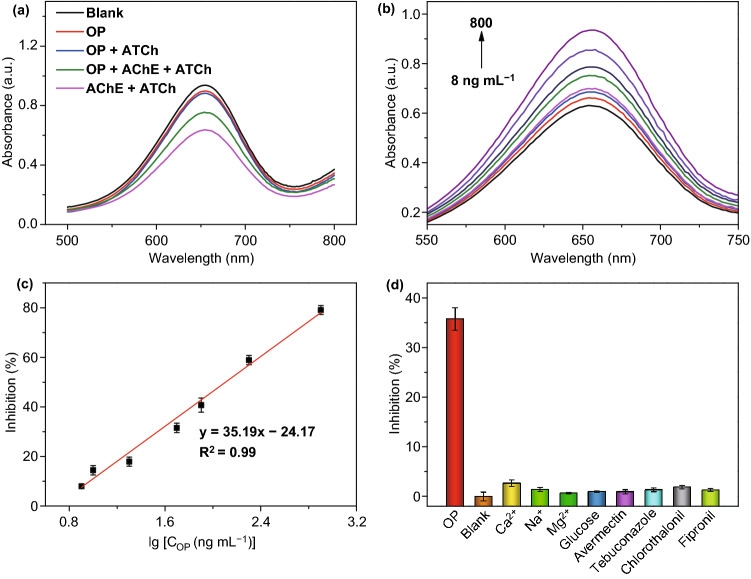
**a** Absorption spectra of OP, OP + ATCh, OP + AChE, and OP + ATCh + AChE in the NO_2_-MIL-101-based biosensor. **b** Absorption spectra of AChE-integrated biosensor in the presence of different amounts of OP. **c** A linear relationship between the concentration of OP and inhibition of AChE activity. **d** Selectivity test of NO_2_-MIL-101-based biosensor in the presence of interfering molecules

Organophosphorus compounds are used as pesticides all over the world. However, they can irreversibly inhibit the AChE in the body and further give rise to damages in the nervous system [56, 57]. Therefore, it is of great importance to trace the concentration of organophosphorus pesticides (OP). Given the inhibition effect of OP, AChE was further integrated into NO_2_-MIL-101-based biosensor to monitor the amounts of paraoxon-ethyl (a typical OP). As shown in Fig. 5a, the introduction of OP showed a negligible influence on the biosensing system. With the increasing amounts of OP, the activity of AChE was inhibited gradually and the biosensor delivered the stronger absorbance signals (Fig. 5b). This biosensor exhibits favorable linear relationships between the inhibition rate and concentration of OP from 8 to 800 ng mL^−1^ with a LOD of 1 ng mL^−1^ (Fig. 5c). Compared with other methods, the NO_2_-MIL-101-based biosensor possesses excellent performance (Table S8). Due to the complexity of the actual testing environment, the specificity, reproductivity and stability are important factors to evaluate the biosensor performance. Here, a series of related experiments demonstrated that the as-prepared biosensor exhibits satisfactory selectivity, reproductivity and stability (Figs. 5d, S11b and S12b). Furthermore, NO_2_-MIL-101-based biosensor was used to determine the concentration of OP in tap water, river water, rice and apple samples. The satisfactory recoveries verified this biosensor holds great application potential in environmental and agricultural analysis (Table S6).

## Conclusions

In summary, rational design and synthesis of the functionalized MOFs were realized by introducing the strong electron-withdrawing (or donating) substituent (–NO_2_ or –NH_2_) into the organic linker, achieving the fine regulation in tuning atomically dispersed metal sites for boosted POD-like activity. As a result, the introduction of –NO_2_ significantly facilitates the POD-like activity of nanozymes and affinity toward H_2_O_2_, while the –NH_2_ is poles apart. Furthermore, theoretical calculations suggest that the adjacent –NO_2_ can not only change the geometry of adsorbed H_2_O_2_* to enhance the binding strength of intermediates, but also optimize the electronic structure of atomically dispersed Fe to promote the adsorption of the intermediate HO* species. Benefiting from these geometric and electronic effects, the NO_2_-MIL-101 possesses the optimal RDS energy change, which can markedly improve POD-like activity. Accordingly, NO_2_-MIL-101 was successfully used to construct biosensor for highly sensitive colorimetric detection of AChE activity and OP with a LOD of 0.14 mU mL^−1^ and 1 ng mL^−1^, respectively. Importantly, the constructed biosensor characterized with satisfactory selectivity, reproductivity and stability, showed potential applications in the analysis of practical samples. This work not only provides a strategy to develop high-efficiency nanozymes by combining the geometric and electronic effects, but also expands the application of nanozymes in biosensing.

## Electronic supplementary material

Below is the link to the electronic supplementary material.

Supplementary material 1 (PDF 574 kb)

## References

[1] Komkova MA, Karyakina EE, Karyakin AA (2018). Catalytically synthesized prussian blue nanoparticles defeating natural enzyme peroxidase. J. Am. Chem. Soc..

[2] Huang Y, Ren J, Qu X (2019). Nanozymes: classification, catalytic mechanisms, activity regulation, and applications. Chem. Rev..

[3] Pott M, Hayashi T, Mori T, Mittl PRE, Green AP, Hilvert D (2018). A noncanonical proximal heme ligand affords an efficient peroxidase in a globin fold. J. Am. Chem. Soc..

[4] Berglund GI, Carlsson GH, Smith AT, Szöke H, Henriksen A, Hajdu J (2002). The catalytic pathway of horseradish peroxidase at high resolution. Nature.

[5] Gao L, Zhuang J, Nie L, Zhang J, Zhang Y (2007). Intrinsic peroxidase-like activity of ferromagnetic nanoparticles. Nat. Nanotechnol..

[6] Wang H, Wan K, Shi X (2019). Recent advances in nanozyme research. Adv. Mater..

[7] Zhang Z, Zhang X, Liu B, Liu J (2017). Molecular imprinting on inorganic nanozymes for hundred-fold enzyme specificity. J. Am. Chem. Soc..

[8] Zhang J, Wu S, Lu X, Wu P, Liu J (2019). Manganese as a catalytic mediator for photo-oxidation and breaking the pH limitation of nanozymes. Nano Lett..

[9] Zhang H, Liang X, Han L, Li F (2018). “Non-naked” gold with glucose oxidase-like activity: a nanozyme for tandem catalysis. Small.

[10] Zhang R, Lua N, Zhang J, Yan R, Li J (2020). Ultrasensitive aptamer-based protein assays based on one-dimensional core-shell nanozymes. Biosens. Bioelectron..

[11] Lu N, Zhang M, Ding L, Zheng J, Zeng C (2017). Yolk–shell nanostructured Fe_3_O_4_@C magnetic nanoparticles with enhanced peroxidase-like activity for label-free colorimetric detection of H_2_O_2_ and glucose. Nanoscale.

[12] Li Z, Yang X, Yang Y, Tan Y, He Y (2018). Peroxidase-mimicking nanozyme with enhanced activity and high stability based on metal–support interactions. Chem. Eur. J..

[13] Cai S, Fu Z, Xiao W, Xiong Y, Wang C, Yang R (2020). Zero-dimensional/two-dimensional Au_x_Pd_100−x_ nanocomposites with enhanced nanozyme catalysis for sensitive glucose detection. ACS Appl. Mater. Interfaces.

[14] Zeng C, Lu N, Wen Y, Liu G, Zhang R (2019). Engineering nanozymes using DNA for catalytic regulation. ACS Appl. Mater. Interfaces.

[15] Jiao L, Xu W, Yan H, Wu Y, Liu C (2019). Fe–N–C single-atom nanozymes for the intracellular hydrogen peroxide detection. Anal. Chem..

[16] Jiao L, Yan H, Wu Y, Gu W, Zhu C, Du D, Lin Y (2019). When nanozymes meet single-atom catalysis. Angew. Chem. Int. Ed..

[17] Wu Y, Wu J, Jiao L, Xu W, Wang H (2020). Cascade reaction system integrating single-atom nanozymes with abundant Cu sites for enhanced biosensing. Anal. Chem..

[18] Huang L, Chen J, Gan L, Wang J, Dong S (2019). Single-atom nanozymes. Sci. Adv..

[19] Cheng N, Li J-C, Liu D, Lin Y, Du D (2019). Single-atom nanozyme based on nanoengineered Fe–N–C catalyst with superior peroxidase-like activity for ultrasensitive bioassays. Small.

[20] Lu M, Wang C, Ding Y, Peng M, Zhang W (2019). Fe–N/C single-atom catalysts exhibiting multienzyme activity and ROS scavenging ability in cells. Chem. Commun..

[21] Zhao C, Xiong C, Liu X, Qiao M, Li Z (2019). Unraveling the enzyme-like activity of heterogeneous single atom catalyst. Chem. Commun..

[22] Jiao L, Wu J, Zhong H, Zhang Y, Xu W (2020). Densely isolated FeN_4_ sites for peroxidase mimicking. ACS Catal..

[23] Ding M, Flaig RW, Jiang H-L, Yaghi OM (2019). Carbon capture and conversion using metal–organic frameworks and MOF-based materials. Chem. Soc. Rev..

[24] Mason JA, Oktawiec J, Taylor MK, Hudson MR, Rodriguez J (2015). Methane storage in flexible metal–organic frameworks with intrinsic thermal management. Nature.

[25] Zhang L, Li L, Hu E, Yang L, Shao K (2019). Boosting ethylene/ethane separation within copper(i)-chelated metal–organic frameworks through tailor-made aperture and specific π-complexation. Adv. Sci..

[26] Lian X, Fang Y, Joseph E, Wang Q, Li J (2017). Enzyme–MOF (metal–organic framework) composites. Chem. Soc. Rev..

[27] Lu W, Wei Z, Gu Z-Y, Liu T-F, Park J (2014). Tuning the structure and function of metal–organic frameworks via linker design. Chem. Soc. Rev..

[28] Li B, Chrzanowski M, Zhang Y, Ma S (2016). Applications of metal-organic frameworks featuring multi-functional sites. Coord. Chem. Rev..

[29] Chen C-X, Wei Z-W, Jiang J-J, Zheng S-P, Wang H-P (2017). Dynamic spacer installation for multirole metal–organic frameworks: a new direction toward multifunctional MOFs achieving ultrahigh methane storage working capacity. J. Am. Chem. Soc..

[30] Wang F, Liu Z, Yang C, Zhong H, Nam G (2020). Fully conjugated phthalocyanine copper metal–organic frameworks for sodium–iodine batteries with long-time-cycling durability. Adv. Mater..

[31] Jiang ZW, Zou YC, Zhao TT, Zhen SJ, Li YF, Huang CZ (2020). Controllable synthesis of porphyrin-based 2D lanthanide metal-organic frameworks with thickness- and metal node-dependent photocatalytic performances. Angew. Chem. Int. Ed..

[32] Xue Z, Liu K, Liu Q, Li Y, Li M (2019). Missing-linker metal-organic frameworks for oxygen evolution reaction. Nat. Commun..

[33] Chambers MB, Wang X, Ellezam L, Ersen O, Fontecave M (2017). Maximizing the photocatalytic activity of metal–organic frameworks with aminated-functionalized linkers: substoichiometric effects in MIL-125-NH_2_. J. Am. Chem. Soc..

[34] Nguyen HL, Vu TT, Le D, Doan TLH, Nguyen VQ, Phan NTS (2017). A titanium–organic framework: engineering of the band-gap energy for photocatalytic property enhancement. ACS Catal..

[35] Zhao M, Yuan K, Wang Y, Li G, Guo J (2016). Metal–organic frameworks as selectivity regulators for hydrogenation reactions. Nature.

[36] Jiao L, Wang Y, Jiang H-L, Xu Q (2018). Metal–organic frameworks as platforms for catalytic applications. Adv. Mater..

[37] Li S, Liu X, Chai H, Huang Y (2018). Recent advances in the construction and analytical applications of metal-organic frameworks-based nanozymes. TrAC Trends Anal. Chem..

[38] Nath I, Chakraborty J, Verpoort F (2016). Metal organic frameworks mimicking natural enzymes: a structural and functional analogy. Chem. Soc. Rev..

[39] Chen W-H, Vázquez-González M, Kozell A, Cecconello A, Willner I (2018). Cu^2+^-modified metal–organic framework nanoparticles: a peroxidase-mimicking nanoenzyme. Small.

[40] Xu W, Jiao L, Yan H, Wu Y, Chen L (2019). Glucose oxidase-integrated metal-organic framework hybrids as biomimetic cascade nanozymes for ultrasensitive glucose biosensing. ACS Appl. Mater. Interfaces.

[41] Kresse G, Furthmüller J (1996). Efficiency of ab initio total energy calculations for metals and semiconductors using a plane-wave basis set. Comput. Mater. Sci..

[42] Perdew JP, Burke K, Ernzerhof M (1996). Generalized gradient approximation made simple. Phys. Rev. Lett..

[43] Kresse G, Furthmüller J (1996). Efficient iterative schemes for ab initio total-energy calculations using a plane-wave basis set. Phys. Rev. B.

[44] Hu Y, Cheng H, Zhao X, Wu J, Muhammad F (2017). Surface-enhanced Raman scattering active gold nanoparticles with enzyme-mimicking activities for measuring glucose and lactate in living tissues. ACS Nano.

[45] Mateo D, Santiago-Portillo A, Albero J, Navalón S, Alvaro M, García H (2019). Long-term photostability in terephthalate metal–organic frameworks. Angew. Chem. Int. Ed..

[46] Wang Y, Wang L, Huang W, Zhang T, Hu X, Perman JA, Ma S (2017). A metal–organic framework and conducting polymer based electrochemical sensor for high performance cadmium ion detection. J. Mater. Chem. A.

[47] Dery S, Kim S, Tomaschun G, Haddad D, Cossaro A (2019). Flexible NO_2_-functionalized N-heterocyclic carbene monolayers on Au (111) surface. Chem. Eur. J..

[48] Jiang B, Duan D, Gao L, Zhou M, Fan K (2018). Standardized assays for determining the catalytic activity and kinetics of peroxidase-like nanozymes. Nat. Protoc..

[49] Wei Z, Villamena FA, Weavers LK (2017). Kinetics and mechanism of ultrasonic activation of persulfate: an in situ EPR spin trapping study. Environ. Sci. Technol..

[50] Liu Y, Jin W, Zhao Y, Zhang G, Zhang W (2017). Enhanced catalytic degradation of methylene blue by α-Fe_2_O_3_/graphene oxide via heterogeneous photo-Fenton reactions. Appl. Catal. B Environ..

[51] Jorfi S, Kakavandi B, Motlagh HR, Ahmadi M, Jaafarzadeh N (2017). A novel combination of oxidative degradation for benzotriazole removal using TiO_2_ loaded on Fe^ii^Fe_2_^iii^O_4_@C as an efficient activator of peroxymonosulfate. Appl. Catal. B Environ..

[52] Deringer VL, Tchougréeff AL, Dronskowski R (2011). Crystal orbital hamilton population (COHP) analysis as projected from plane-wave basis sets. J. Phys. Chem. A.

[53] Zhang J, Zheng W, Jiang X (2018). Ag^+^-gated surface chemistry of gold nanoparticles and colorimetric detection of acetylcholinesterase. Small.

[54] Wu Y, Jiao L, Luo X, Xu W, Wei X (2019). Oxidase-like Fe–N–C single-atom nanozymes for the detection of acetylcholinesterase activity. Small.

[55] Niu X, Shi Q, Zhu W, Liu D, Tian H (2019). Unprecedented peroxidase-mimicking activity of single-atom nanozyme with atomically dispersed Fe–Nx moieties hosted by MOF derived porous carbon. Biosens. Bioelectron..

[56] Wu Y, Jiao L, Xu W, Gu W, Zhu C, Du D, Lin Y (2019). Polydopamine-capped bimetallic AuPt hydrogels enable robust biosensor for organophosphorus pesticide detection. Small.

[57] Jin R, Kong D, Zhao X, Li H, Yan X (2019). Tandem catalysis driven by enzymes directed hybrid nanoflowers for on-site ultrasensitive detection of organophosphorus pesticide. Biosens. Bioelectron..

