# Supplementary material for: Tuning Atomically Dispersed Fe Sites in Metal–Organic Frameworks Boosts Peroxidase-Like Activity for Sensitive Biosensing
Source: Nanomicro Lett. 2020 Sep 23;12:184. doi: 10.1007/s40820-020-00520-3 (PMC7770903; doi:10.1007/s40820-020-00520-3)
Supplement: Supplementary file 1 — Supplementary material 1 (PDF 574 kb) [file 40820_2020_520_MOESM1_ESM.pdf]

Supporting Information for

Tuning Atomically Dispersed Fe Sites in Metal-Organic Frameworks Boosts Peroxidase-Like Activity for Sensitive Biosensing

Weiqing Xu[1], Yikun Kang[2], Lei Jiao[1], Yu Wu[1], Hongye Yan[1], Jinli Li[1], Wenling Gu[1], Weiyu Song[2,] *, Chengzhou Zhu[1,] *

[1]Key Laboratory of Pesticide and Chemical Biology of Ministry of Education, International Joint Research Center for Intelligent Biosensing Technology and Health, College of Chemistry, Central China Normal University, Wuhan, 430079, People's Republic of China

[2]State Key Laboratory of Heavy Oil Processing, China University of Petroleum, Beijing 102249, People's Republic of China

Weiqing Xu and Yikun Kang contributed equally to this work

*Correponding authors. E-mail: songwy@cup.edu.cn (Weiyu Song); czzhu@mail.ccnu.edu.cn (Chengzhou Zhu)

S1 Instruments

Transmission electron microscopy (TEM) experiments were performed using a FEI Talos F200x (super-x). The element contents were obtained by inductively coupled plasma optical emission (ICP-OES) spectrometry (Agilent 8800). Powder X-ray diffraction (XRD) patterns were carried using a Tensor 27. The functional groups were analyzed using a NEXUS870 FT-IR spectrometer. X-ray photoelectron spectroscopy (XPS) measurements were used by a VG Multilab 2000 (Thermo Fisher, USA). Electron paramagnetic resonance (EPR) measurements were obtained by an EMXmicro-6/1 (Bruker, Germany). Ultrapure water was obtained from a Milli-Q purification system (Millipore, MA, USA). All the UV-vis and fluorescence spectra were obtained from a multimode reader (Tecan Spark, Switzerland).

S2 Experimental Section

S2.1 Preparation of MIL-101

$FeCl_3$•$6H_2O$ (675 mg, 2.5 mmol) and terephthalic acid (206 mg, 1.25 mmol) were dissolved in 15 mL N,N-dimethylformamide (DMF) and then the solution was vigorously stirred for 1 h. After that, the mixture transported to a Teflon-lined autoclave for 15 hours at 110 °C. The orange products were washed with DMF and ethanol three times. Finally, the solvent was dried overnight at 80 °C to obtain the powder.

S2.2 Preparation of NO_2-MIL-101

$FeCl_3 \cdot 6H_2O$ (675 mg, 2.5 mmol) and 2-nitroterephthalic acid (264 mg, 1.25 mmol) were dissolved in 15 mL DMF and then the solution was vigorously stirred for 1 h. After that, the mixture transported to a Teflon-lined autoclave for 12 h at 110 °C. The orange products were washed with DMF and ethanol three times. Finally, the solvent was dried overnight at 80 °C to obtain the powder.

S2.3 Preparation of NH_2-MIL-101

$FeCl_3 \cdot 6H_2O$ (675 mg, 2.5 mmol) and 2-aminoterephthalic acid (226 mg, 1.25 mmol) were dissolved in 15 mL DMF and then the solution was vigorously stirred for 1 h. After that, the mixture transported to a Teflon-lined autoclave for 12 h at 110 °C. The orange products were washed with DMF and ethanol three times. Finally, the solvent was dried overnight at 80 °C to obtain the powder.

S2.4 Specific Activity of Nanozymes

The specific activity (SA), which is defined as activity units per milligram of nanozyme, was evaluated in different concentrations of nanozymes [S1]. The nanozyme activity (units) was calculated using Eq. (S1):

$$b_{nanozyme} = \frac{V \times (\frac{\Delta A}{\Delta t})}{\varepsilon \times l} \qquad \text{(S1)}$$

$b_{nanozyme}$ is the catalytic activity of nanozyme expressed in units. V is the total volume of the reaction solution (μL); ε is the molar absorption coefficient of the colorimetric TMB (39,000 (M^{-1} cm^{-1}). l is the path length of light traveling in the cuvette (cm); A is the absorbance value; and ΔA/Δt is the initial rate of change in absorbance at 652 nm min^{-1}.

Calculate the SA of the nanozyme (U mg^{-1}) by nanozyme: $a_{nanozyme} = b_{nanozyme}/[m]$. Where $a_{nanozyme}$ is the SA expressed in units per milligram (U mg^{-1}) nanozymes, and [m] is the nanozyme weight (mg) of each assay.

S2.5 Verification of Intermediate (•OH)

The blue methylene blue (MB) could be degraded to the colourless products in the presence of •OH. Therefore, MB was usually employed to verify the existence of •OH by colorimetric assay [S2]. The nanozymes (1 mg mL^{-1}, 100 μL) were added into the HAc-NaAc buffer (0.1 M, pH 3.0, 1 mL) containing H_2O_2 (1 M, 1 mL) and MB (1 mM, 100 μL), respectively. Then, the absorbance of the reaction solution was monitored after 1.5 h.

S2.6 Computation Details

For the calculation of the energy change diagram of reaction, we followed the mechanism in the acidic environment as Eqs. S2-S5:

$$H_2O_2 + * \rightarrow H_2O_2* \qquad \text{(S2)}$$

$$H_2O_2* \rightarrow OH* + \bullet OH \qquad \text{(S3)}$$

$$OH^* + H^+ + e^- \rightarrow H_2O^* \quad (S4)$$

$$H_2O^* \rightarrow H_2O + * \quad (S5)$$

S2.7 Interference Study for AChE Detection

A series of proteins were chosen to measure the anti-interference ability of the NO_2-MIL-101 based biosensor. Instead of AChE, 50 µL 10 µg mL^{-1} HRP (horseradish peroxidase), LAC (Laccase), GOx (Glucose oxidase), INV (Invertase), and BSA (bovine serum albumin) were added into the reaction system respectively. The absorbance values at 652 nm were recorded to evaluate the interference effect.

S2.8 Recovery Test for AChE

Human serum samples diluted 100 times for the recovery test of AChE. Then different concentration AChE were added into the diluted serum samples for the recovery tests.

S2.9 Colorimetric Detection of Paraoxon-ethyl

AChE (50 mU mL^{-1}, 50 µL, pH 7.4) and different concentration paraoxon-ethyl (50 µL) were incubated for 30 min 37 °C. The ATCh (10 mM, 30 µL, pH 7.4) was introduced into this system for another 30 min. The subsequent processes were the same as the procedure of AChE detection and the absorbance of this system was named as A_1. Two parallel experiments were carried out at the same time. For one paraoxon-ethyl was not added into the reaction system (A). For another, the paraoxon-ethyl and ATCh were not added into the reaction system (A_0). The inhibition rate was used to measure the amounts paraoxon-ethyl, and the inhibition rate was calculated by Eq. S6:

$$\text{Inhibition (\%)} = \frac{(A_1\text{-}A)}{(A_0\text{-}A)} \times 100\% \quad (S6)$$

S2.10 Interference Study for Paraoxon-ethyl Detection

A series of interference substances were chosen to measure the anti-interference ability of the NO_2-MIL-101 based biosensor. Instead of paraoxon-ethyl, 50 µL 1 µg mL^{-1} Na^+, Ca^{2+}, Mg^{2+}, glucose and 50 µL 100 ng mL^{-1} avermectin, tebuconazole, chlorothalonil, fipronil were added into the reaction system respectively. The absorbance values at 652 nm were recorded to evaluate the interference effect.

S2.11 Recovery Test for Paraoxon-ethyl

Tap and river water samples were spiked with paraoxon-ethyl after a filtration. Besides, the rice and apple samples were washed with ultrapure water and then dried at room temperature. Then, 3 g of sample was placed into an ultrasonic bath for 5min before being centrifuged for 10 min (12,000 rpm). Later, a different amount of OP was added into the real samples. The concentration of the real samples was determined by the calibration curve.

S3 Supplementary Figures and Tables

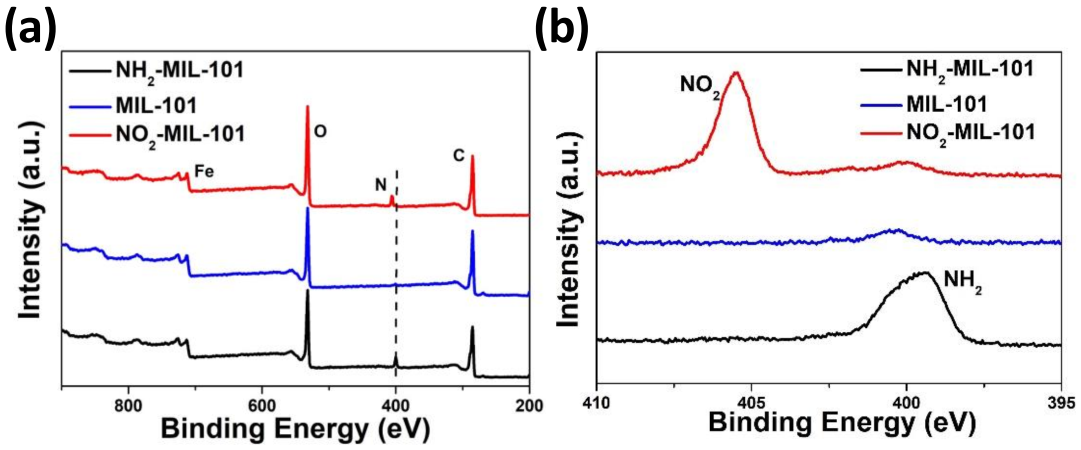

Fig. S1 a Full range XPS and **b** N 1s spectra of different nanozymes

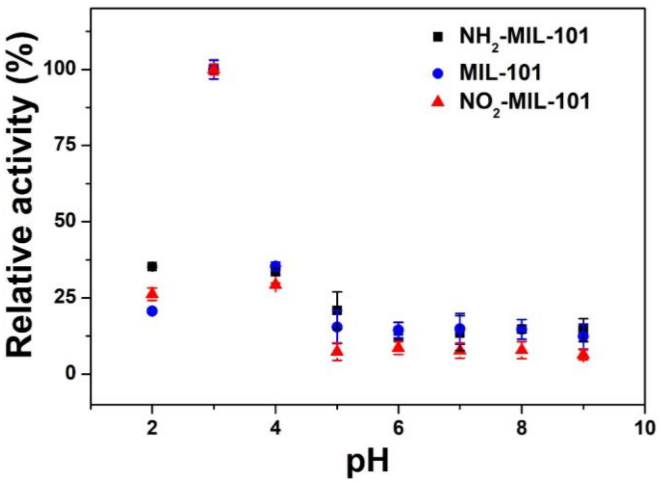

Fig. S2 Effect of pH on the relative activity of different nanozymes

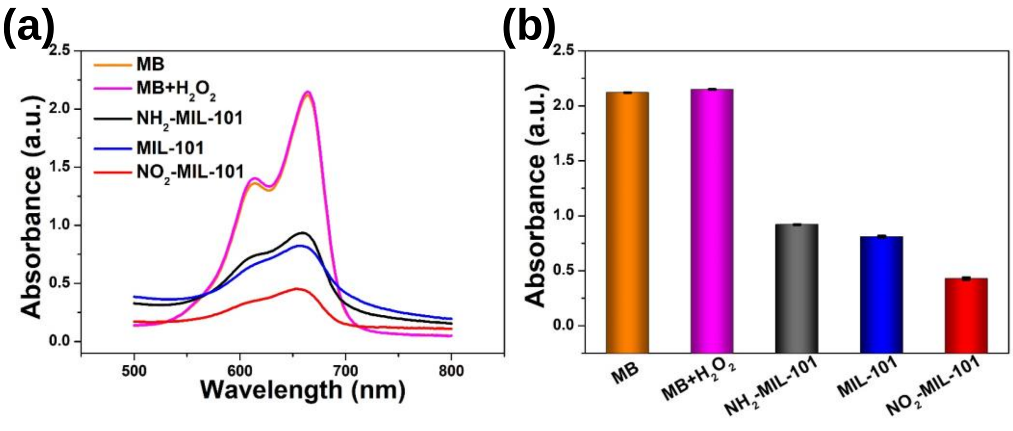

Fig. S3 a Absorption spectra and **b** absorbance values (664 nm) of different nanozymes in H_2O_2/MB solution

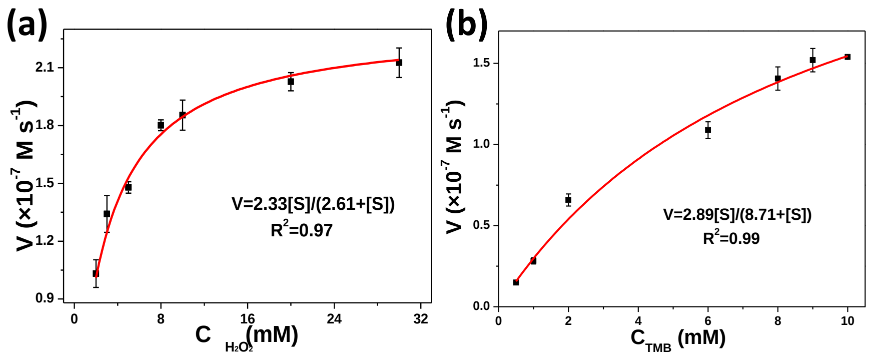

Fig. S4 Steady-state kinetic analyses for NH_2-MIL-101 using Michaelis-Menten equation as the non-linear least-squares regression. **a** Concentration of H_2O_2 was 0.5 M and the TMB concentration was varied. **b** Concentration of TMB was 10 mM and the H_2O_2 concentration was varied

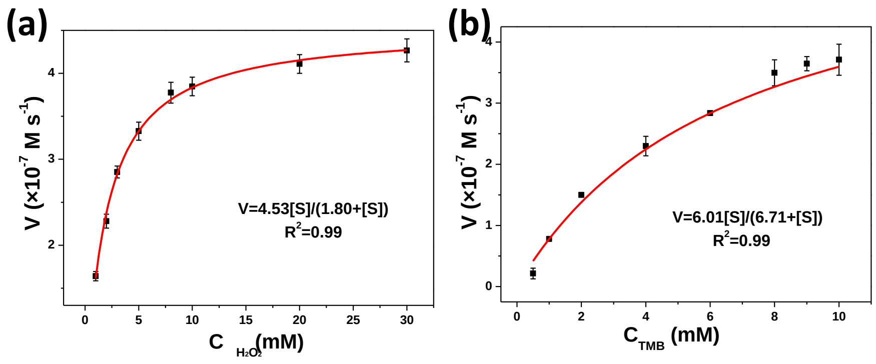

Fig. S5 Steady-state kinetic analyses for MIL-101 using Michaelis-Menten equation as the non-linear least-squares regression. **a** Concentration of H_2O_2 was 0.5 M and the TMB concentration was varied. **b** Concentration of TMB was 10 mM and the H_2O_2 concentration was varied

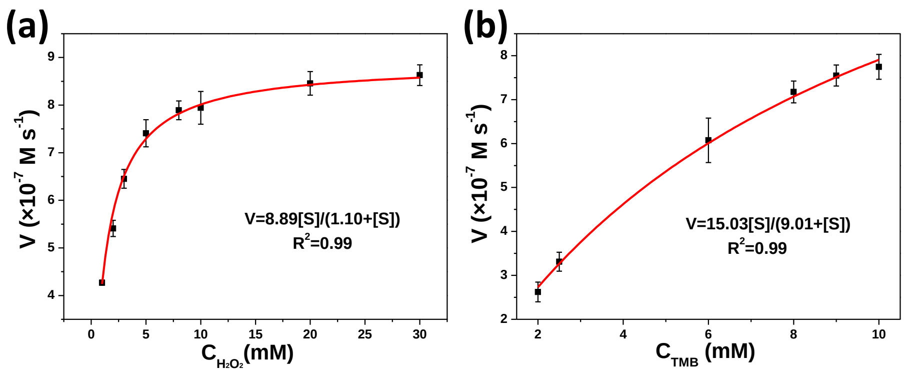

Fig. S6 Steady-state kinetic analyses for NO_2-MIL-101 using Michaelis-Menten equation as the non-linear least-squares regression. **a** Concentration of H_2O_2 was 0.5 M and the TMB concentration was varied. **b** Concentration of TMB was 10 mM and the H_2O_2 concentration was varied

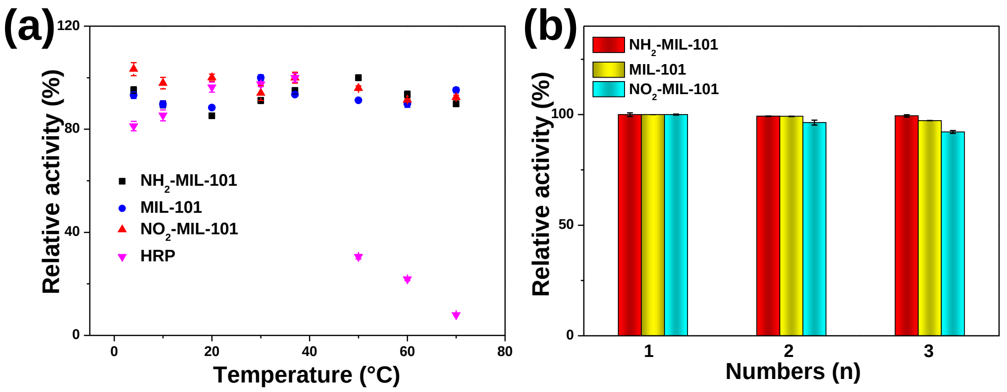

Fig. S7 a Effects of temperature on the relative activity of NH_2-MIL-101, MIL-101, NO_2-MIL-101, and HRP. **B** Reproducibility of the resultant MOFs

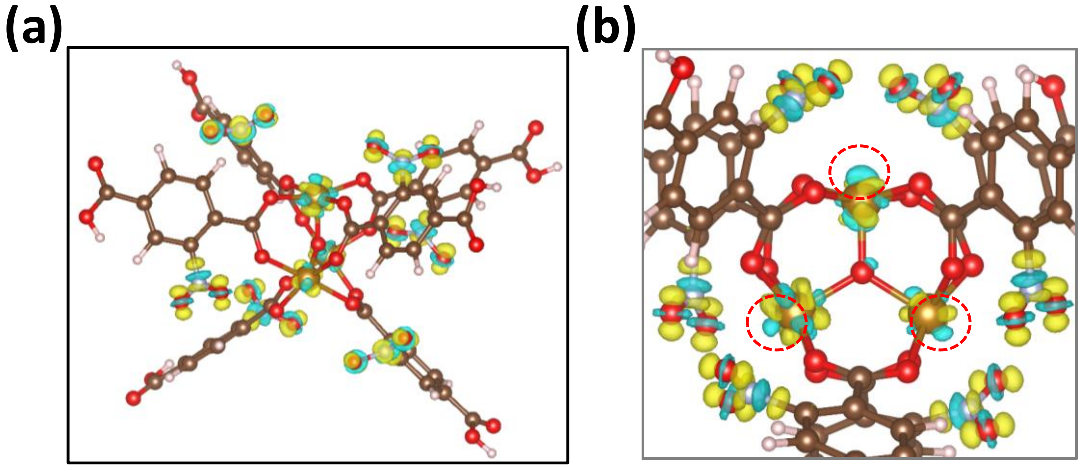

Fig. S8 a Charge density difference of NO_2-MIL-101 between MIL-101 and nitro ligands. **b** Side view of charge density difference of NO_2-MIL-101. The charge decrease of the dangling bond marked with red line at active site

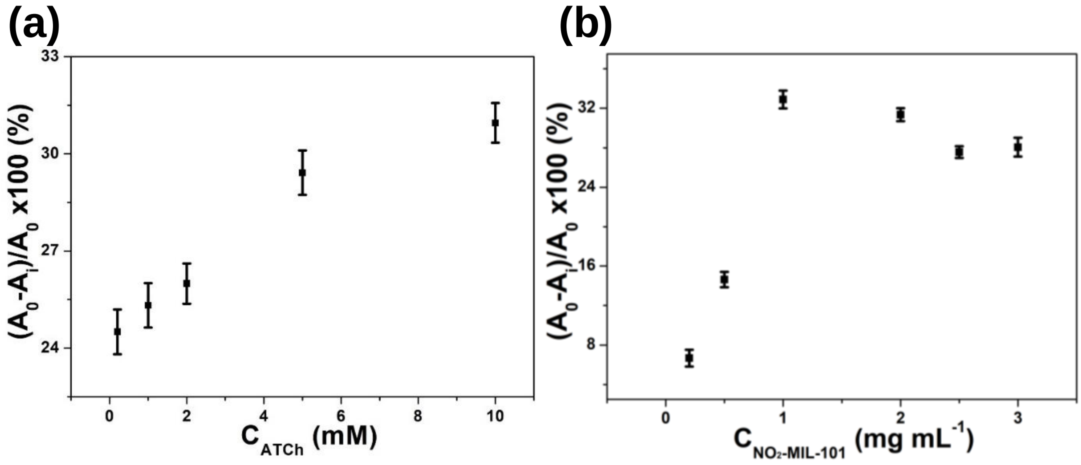

Fig. S9 Influence of the **a** ATCh and **b** NO_2-MIL-101 concentration (the original concentration) on the performance of NO_2-functionalized MIL-101-based biosensor

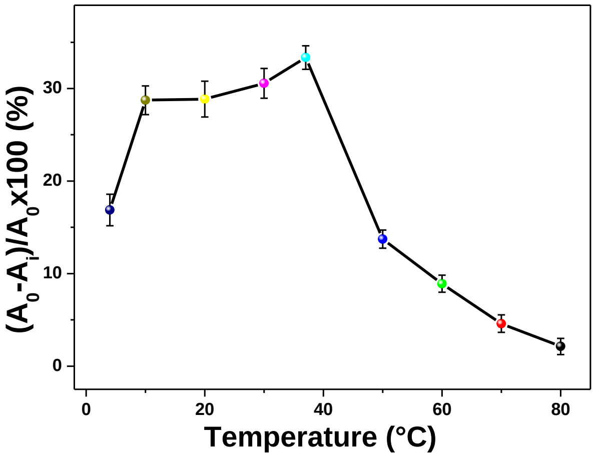

Fig. S10 Influence of the temperature on the performance of NO_2-functionalized MIL-101-based biosensor

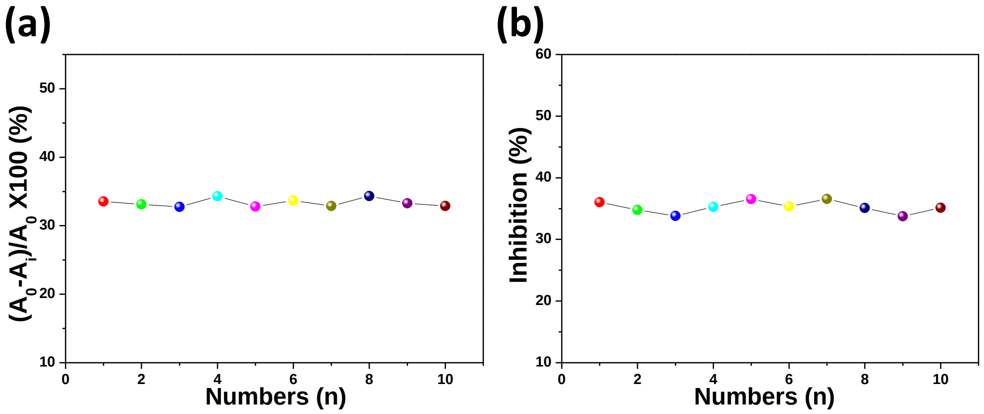

Fig. S11 Reproducibility of NO_2-MIL-101-biosensor for the detection of **a** AChE and **b** OP

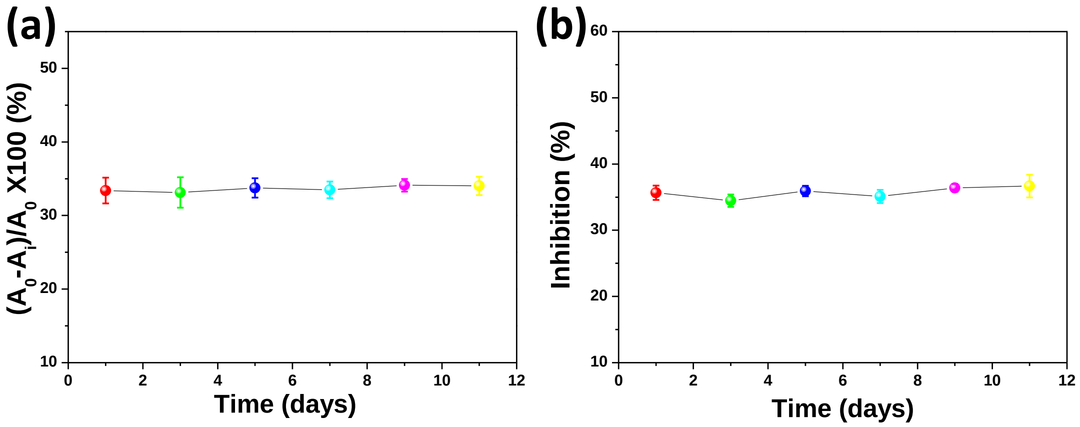

Fig. S12 Stability of NO_2-MIL-101-biosensor for the detection of **a** AChE and **b** OP

Table S1 Content of Fe in different nanozymes

Nanozymes	Mass ratio (mg mg^{-1})	Atomic concentration (%)
NH_2-MIL-101	0.13	4.54
MIL-101	0.16	4.69
NO_2-MIL-101	0.15	4.15

The mass ratio was obtained by ICP-OES, and the atomic concentration was obtained by XPS.

Table S2 Maximum reaction rate (V_{max}) and Michaelis constant (K_m) of different nanozymes

Nanozymes	Substrate	V_{max} ($\times 10^{-7}$ M s^{-1})	K_m ($\times 10^{-3}$ M)
NH_2-MIL- 101	TMB	2.89	8.71
	H_2O_2	2.33	2.61
MIL- 101	TMB	6.01	6.71
	H_2O_2	4.53	1.80
NO_2-MIL- 101	TMB	15.03	9.01
	H_2O_2	8.89	1.10

Table S3 Integration of the project electronic density of states (PDOS) to Fermi level of each split Fe 3d orbit on MIL-101, NH_2-MIL-101, and NO_2-MIL-101

Split Fe 3d orbit	MIL-101	NH_2-MIL-101	NO_2-MIL-101
d_{xy}	3.771	3.439	3.434
d_{yz}	3.464	3.621	3.550
d_{xz}	3.185	3.294	3.596
d_{z^2}	3.880	3.812	3.337
$d_{x^2-z^2}$	3.588	3.590	3.573

Table S4 ICOHP of HO*-Fe bond in MIL-101, NH_2-MIL-101, and NO_2-MIL-101

ICOHP	MIL-101	NH_2-MIL-101	NO_2-MIL-101
HO*-Fe	-4.11	-4.13	-4.80

Table S5 Recoveries of AChE in serum samples (n=3)

Samples	Spiked concentration	Measured concentration	Recovery (%)	RSD (%)
Serum	10 mU mL^{-1}	9.6 mU mL^{-1}	96.0	2.85
	50 mU mL^{-1}	51.5 mU mL^{-1}	102.1	4.64

Table S6 Recoveries of OP in tap and river water, rice and apple (n=3)

Samples	Spiked concentration	Measured concentration	Recovery (%)	RSD (%)
Tap water	50 ng mL^{-1}	47.3 ng mL^{-1}	94.5	2.84
	100 ng mL^{-1}	91.4 ng mL^{-1}	91.4	3.79
River water	50 ng mL^{-1}	47.71 ng mL^{-1}	95.42	4.84
	100 ng mL^{-1}	97.72 ng mL^{-1}	97.72	2.63
Rice	50 ng mL^{-1}	48.98 ng mL^{-1}	97.96	4.07
	100 ng mL^{-1}	99.08 ng mL^{-1}	99.08	4.11
Apple	50 ng mL^{-1}	52.78 ng mL^{-1}	105.56	3.42
	100 ng mL^{-1}	107.15 ng mL^{-1}	107.15	2.93

Table S7 Comparison of different biosensors for the detection of AChE

Biosensor	Method	Linear range (mU mL^{-1})	LOD (mU mL^{-1})	Refs.
Poly{1,4-phenylene-[9,9 -bis(4-phenoxybutylsulfonate)]fluorene-2,7-diyl} (PFP-SO_3^-)	Fluorescent	-	50	[S3]
PAA-CeO_2	Fluorescent	0.263 - 50	0.263	[S4]
AuNCs-Cu^{2+}	Fluorescent	0.05 - 2.5	0.05	[S5]
Citrate-CeO_2	Colormetric	0 - 1400	3.5	[S6]
Au@PDA NPs hydrogel	Colorimetric	2.5 - 25	0.9	[S7]
NO_2-MIL-101	Colorimetric	0.2 - 50	0.1	This work

PAA: Polyacrylic Acid, PDA: Polydopamine.

Table S8 Comparison of different paraoxon-ethyl biosensors

Biosensor	Method	Linear range (ng mL^{-1})	LOD (ng mL^{-1})	Refs.
PAA-CeO_2	Fluorescent	100 - 1000	27	[S4]
AChE-MnO_2-TMB	Colorimetric	1 - 100	1.0	[S8]
CDs/DNTB/ATCh/AChE	Colorimetric or fluorescent	1 - 1000	0.4	[S9]
Fe-N-C SAzymes	Colorimetric	100 - 10000	0.97	[S10]
AChE/AuPt-PDA	Electrochemistry	0.5 - 1000	0.185	[S11]
NO_2-MIL-101	Colorimetric	8 - 800	1.0	This work

Supplementary References

[S1] B. Jiang, D. Duan, L. Gao, M. Zhou, K. Fan et al., Standardized assays for determining the catalytic activity and kinetics of peroxidase-like nanozymes. Nat. Protoc. **13**, 1506-1520 (2018). https://doi.org/10.1038/s41596-018-0001-1

[S2] Z. Wang, Y. Zhang, E. Ju, Z. Liu, F. Cao et al., Biomimetic nanoflowers by self-assembly of nanozymes to induce intracellular oxidative damage against hypoxic tumors. Nat. Commun. **9**, 3334 (2018). https://doi.org/10.1038/s41467-018-05798-x

[S3] F. Feng, Y. Tang, S. Wang, Y. Li, D. Zhu, Continuous fluorometric assays for acetylcholinesterase activity and inhibition with conjugated polyelectrolytes. Angew. Chem. Int. Ed. **46**, 7882-7886 (2007). https://doi.org/10.1002/ange.200701724

[S4] S.-X.Zhang, S.-F. Xue, J. Deng, M. Zhang, G. Shi, T. Zhou, Polyacrylic acid-coated cerium oxide nanoparticles: an oxidase mimic applied for colorimetric assay to organophosphorus pesticides. Biosens. Bioelectron. **85**, 457-463 (2016). https://doi.org/10.1016/j.bios.2016.05.040

[S5] J. Sun, X. Yang, Gold nanoclusters–Cu^{2+} ensemble-based fluorescence turn-on and real-time assay for acetylcholinesterase activity and inhibitor screening. Biosens. Bioelectron. **74**, 177-182 (2015). https://doi.org/10.1016/j.bios.2015.06.013

[S6] H. Cheng, S. Lin, F. Muhammad, Y.-W. Lin, H. Wei, Rationally modulate the oxidase-like activity of nanoceria for self-regulated bioassays. ACS Sens. **1**, 1336-1343 (2016). https://doi.org/10.1021/acssensors.6b00500

[S7] J. Zhang, L. Mou, X. Jiang, Hydrogels incorporating Au@ polydopamine

nanoparticles: robust performance for optical sensing. Anal. Chem. **90**, 11423-11430 (2018) https://doi.org/10.1021/acs.analchem.8b02459

[S8] X. Yan, Y. Song, X. Wu, C. Zhu, X. Su, D. Du, Y. Lin, Oxidase-mimicking activity of ultrathin MnO_2 nanosheets in colorimetric assay of acetylcholinesterase activity. Nanoscale **9**, 2317-2323 (2017). https://doi.org/10.1039/C6NR08473G

[S9] H. Li, X. Yan, G. Lu, X. Su, Carbon dot-based bioplatform for dual colorimetric and fluorometric sensing of organophosphate pesticides. Sens. Actuat B-Chem. **260**, 563-570 (2018). https://doi.org/10.1016/j.snb.2017.12.170

[S10] Y. Wu, L. Jiao, X. Luo, W. Xu, X. Wei et al., Oxidase-like Fe-N-C single-atom nanozymes for the detection of acetylcholinesterase activity. Small **15**, 1903108 (2019). https://doi.org/10.1002/smll.201903108

[S11] Y. Wu, L. Jiao, W. Xu, W. Gu, C. Zhu, D. Du, Y. Lin, Polydopamine-capped bimetallic AuPt hydrogels enable robust biosensor for organophosphorus pesticide detection. Small **15**, 1900632 (2019). https://doi.org/10.1002/smll.201900632
